# Young Lungs at Play: Preventing Children’s Exposure to Secondhand Smoke in Outdoor Play Areas in a Steps to a HealthierNY County

**Published:** 2007-09-15

**Authors:** Melissa Jacobson, Devon Kessler, Una Diffley, Shelley Chanler, Hailey Reid, Joan Facelle, Allan R. Beers

**Affiliations:** Rockland County Department of Health, Ms Jacobson is the Project Coordinator of the Steps to a HealthierNY program in Rockland County, NY; Rockland County Department of Health, Pomona, New York; Rockland County Department of Health, Pomona, New York; Rockland County Department of Health, Pomona, New York; Rockland County Department of Health, Pomona, New York; Rockland County Department of Health, Pomona, New York; Rockland County Division of Environmental Resources, Pomona, New York

## Introduction

The New York State Clean Indoor Air Act (Public Health Law, Article 13-E) restricts tobacco use at nearly all workplaces including school grounds (1). However, no state regulations exist prohibiting tobacco use in outdoor public areas where children play. Adult smoking negatively impacts children by exposing them to secondhand smoke, increasing their potential to choke or burn themselves with lit cigarette butts, and encouraging a negative health behavior (i.e., by seeing adults smoke) (2). In addition, cigarette butts are the most littered item in the world, causing parks and playgrounds to look dirty and incurring clean-up costs for taxpayers (3).

Young Lungs at Play, developed by Steps to a HealthierNY and the Rockland County Division of Environmental Resources, is a successful and easy-to-implement public health campaign that has a substantial impact in the community (4). The goal of the campaign is to encourage local municipalities and organizations to pass regulations banning smoking in outdoor places where children play and to post signs referencing the Young Lungs at Play campaign in these tobacco-free areas. Another goal of this initiative is to increase the visibility of public health prevention and antismoking messages through the large, appealing signs posted in tobacco-free areas. The campaign has been successful in meeting its goals in many communities and has generated interest among additional organizations and municipalities in creating tobacco-free outdoor play spaces.

The campaign addresses factors contributing to smoking on multiple levels of the socioecological model, including political, interpersonal, and environmental factors. In studying the successes of similar initiatives around the nation, we found that, to be most effective, municipalities must agree to adopt formal laws, ordinances, policies, or resolutions. Young Lungs at Play seeks to address interpersonal factors leading to children's beginning to smoke by changing community norms about smoking and by limiting children's exposure to adults who smoke. It also strives to provide an environment free from smoke and discarded cigarette butts and to increase antismoking messages in the environment through the posting of Young Lungs at Play signs throughout the community.

This article describes the implementation of Young Lungs at Play in Rockland County, New York, a geographically small, suburban county outside of New York City. Rockland County is one of four New York State counties receiving funds from Steps to a HealthierUS, an initiative of the United States Department of Health and Human Services administered by the Centers for Disease Control and Prevention (5). Steps to a HealthierNY is an innovative chronic disease prevention program that employs a community-based and integrated approach to improving physical inactivity, poor nutrition, and tobacco-use prevention to reduce the burden of obesity, diabetes, and asthma.

The smoking rate in Rockland County is 14.1% (6), significantly lower than the state's rate of 20.5% and only slightly above the *Healthy People 2010* target of 12.0% (7). However, the population of Rockland County is diverse, and data suggest that large disparities in smoking rates exist within the county (8). The approach to reducing environmental exposure to tobacco used in the Young Lungs at Play campaign is likely to be well-accepted, regardless of the current attitudes toward smoking in a community, as the best way to begin changing attitudes and norms regarding smoking in places where children are affected. The campaign has now been implemented in three other New York counties with much higher smoking rates and differing attitudes toward smoking. In addition, The Community Survey conducted in Rockland County in May of 2006 showed that, even among respondents who smoke, the majority (51%) indicated they would support a ban on smoking in outdoor places where children play, whereas only a third (36%) of respondents who smoke indicated they would support a ban on smoking in other outdoor public areas (8).

The total cost of the Young Lungs at Play initiative to date is approximately $18,000, of which partnering organizations contributed $11,843. The money invested in the program resulted in the posting of 449 signs indicating tobacco-free zones in 148 outdoor play areas, including municipal play areas (e.g., parks, pools, ball fields), schools, childcare centers, faith-based organizations, day camps, and housing complexes. The program was first implemented countywide in June of 2006 and is ongoing. The program steps are listed in the Table and described here in detail for other communities that want to implement Young Lungs at Play.

### Step 1: Assessing community interest

The first step is to assess community interest in regulation that prohibits smoking in outdoor public spaces where children play, such as playgrounds, fields, and parks. To determine the community's readiness to participate in the initiative and persuade municipalities to participate, some form of survey or focus group should be conducted. The Community Survey has been conducted annually since 2001 via telephone by the Rockland County Department of Health using CATI (Computer Aided Technology, Inc, Buffalo Grove, Illinois) to track attitudes, knowledge, and smoking behaviors. The interviews last approximately 11 minutes and are conducted in English and Spanish. A cross-section sample of 1000 households in Rockland County, New York, is generated using a random-digit–dial method, and participants are interviewed by members of a professional survey research and marketing company.  The results of the Community Survey conducted in 2006 suggested that the majority of county residents were in favor of outdoor smoking bans, particularly in places where children play (8). Selected data from the survey showed that

- 96.5% of people surveyed indicated that they thought secondhand smoke is harmful;
- 68.8% of people surveyed supported regulation that prohibits smoking in outdoor public spaces where children play, such as playgrounds, fields, and parks;
- 56.3% of respondents indicated they would support regulation that prohibits smoking in other outdoor public spaces, such as walking trails, pools, or picnic areas.

### Step 2: Involving policy makers and community members

The second step of implementing the program is to invite policy makers to participate and to provide education about the harmful effects of smoking in places where children play. Attending a town or village board meeting would be helpful, as would providing decision makers with a sample tobacco-free park resolution (9). Educate decision makers about the negative effects of adults smoking in places where children play, and let them know that

- discarded cigarettes cost taxpayers money (3);
- children who see adults smoking in family-friendly places, such as parks and playgrounds, view the behavior as acceptable (9);
- the community supports legislation prohibiting smoking in outdoor spaces.

Invitational materials should also describe the other benefits municipalities would receive by participating in the program. These materials include educational brochures, free signage to let residents know about the tobacco-free play areas, and public recognition through the media. For people interested in promoting the tobacco-free areas, staff from the health department can provide a community awareness event for residents.

### Step 3: Developing the signs

**Figure.**
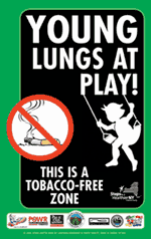
Young Lungs at Play campaign sign, Rockland County, New York, 2006.

Step three of the process is to develop signs to be posted in the tobacco-free areas and pilot test them with community leaders. The signs are made of metal, are weather-resistant, measure 12 in by 18 in, and are in full color. The signs were created around a graphic of a girl on a swing developed by the Tobacco Control Section of the California Department of Health Services (10). Each municipality can customize the signs by including a town or village logo and can request as many signs as needed. Funds for sign production (approximately $20 per sign) were contributed by the POW'R (Putnam, Orange, Westchester, and Rockland counties) Against Tobacco Coalition. The artwork and graphics can be used by other communities replicating the Young Lungs at Play campaign. (Credit for the artwork should be given to the Tobacco Control Section of the California Department of Health Services and the Rockland County Department of Health.)

### Step 4: Holding an opening event

The next step is to determine places that are ready to pass a resolution and invite these community members to a kick-off event with local media and officials at which they will receive the signs to post in their areas. The campaign had its first success in November 2005 when the county of Rockland passed a resolution to create a tobacco-free playground area at Haverstraw Bay Park, the most used of the county's park facilities and the only county park with a playground. In partnership with the county of Rockland's Office of the County Executive, the Rockland County Department of Health, the Rockland County Division of Environmental Resources, and the POW'R Against Tobacco Coalition, all municipalities in Rockland were invited in the spring of 2006 to join the county of Rockland in the Young Lungs at Play campaign. Of the five towns and 19 villages in Rockland County, five municipalities (i.e., the towns of Orangetown and Stony Point and the villages of Nyack, Pomona, and Sloatsburg) adopted a resolution in support of Young Lungs at Play. 

### Step 5: Encouraging other communities to join the campaign

The next step in the process is to contact other municipalities, inform them of the successes of these early adopters, and encourage them to pass their own resolutions. After the kick-off event, other municipalities were again invited to participate in the program and offered assistance. In addition, an ongoing "Join Us" postcard campaign, in which county residents encourage their local municipalities to adopt Young Lungs at Play, is in place. As a result, an additional seven municipalities (i.e., the towns of Clarkstown, Haverstraw, and Ramapo and the villages of Haverstraw, Montebello, Suffern, and Wesley Hills) have joined the campaign.

The program was expanded to include other organizations with outdoor pools or play areas. To date, 15 childcare centers, 11 apartment and condominium complexes, a day camp, and a church have joined the campaign. As a direct result of the program's implementation, three school districts in Rockland County (with a total of 27 elementary schools and one middle school) and three additional elementary schools are posting the signs, increasing the visibility of antismoking messages in the county.

### Step 6: Promoting the efforts of participating communities

The next step is to promote the efforts of the participating communities using, for example, an advertisement in the local newspaper or other media outlet. To recognize communities that adopted resolutions in support of tobacco-free outdoor play areas, "thank-you" advertisements have been placed in four print media outlets. An ongoing campaign in which residents sign "thank-you" postcards that are sent to the town or village board is under way. These efforts are done both to educate the community about the initiatives and the areas affected and to promote the communities that have taken a step toward protecting children from the harmful effects of tobacco.

### Step 7: Continuing to promote the campaign

Finally, continuing to promote the campaign and offering materials and support to interested communities or organizations is important to the continued success of the program. In addition to encouraging municipalities and housing complexes to create tobacco-free play areas, the campaign is now being promoted to day camps in the county. The focus of this initiative is expanding to include not only outdoor play spaces but also other outdoor spaces such as bus shelters.

The campaign is being implemented in three other counties in New York State: Broome, Chatauqua, and Jefferson. These counties are diverse in geography and population and have been successful in promoting the Young Lungs at Play campaign, despite higher smoking rates (Broome County, 24.3%; Chatauqua County, 23.6%; Jefferson County, 22.4%) (6). Although these counties are in the beginning stages of implementing the campaign, they have created many tobacco-free play areas and continue to recruit municipalities and organizations within their communities.

## Conclusion

For a relatively low investment of time and money, the Young Lungs at Play campaign has been successful at increasing the visibility of antismoking messages and modeling good health habits for children. We hope that in the future, the Young Lungs at Play signs will be posted in outdoor play areas throughout the country and will serve as a reminder that smoking is not acceptable or allowed where children play.

## Figures and Tables

**Table:** Steps to Implementing Young Lungs at Play, Rockland County, New York, June 2006

Step No.	Description
1	Assess community interest in regulation that prohibits smoking in outdoor public spaces where children play, such as playgrounds, fields, and parks.
2	Invite policy makers to participate in the program and provide education about the harmful effects of smoking in places where children play.
3	Develop signs to be posted in the tobacco-free areas and pilot test them with community leaders.
4	Determine communities that are ready to pass a resolution, and invite members to a kick-off event with local media and officials at which they will receive the signs to post in their areas.
5	Contact other municipalities and inform them of the successes of these early adopters. Encourage them to pass their own resolutions.
6	Promote the efforts of the participating communities through an advertisement in the local newspaper or other media outlet.
7	Continue to promote the campaign and offer materials and support to interested communities or organizations.
